# Culture of equine intestinal epithelial stem cells after delayed tissue storage for future applications

**DOI:** 10.1186/s12917-022-03552-6

**Published:** 2022-12-23

**Authors:** Amy Stieler Stewart, Cecilia R. Schaaf, Brittany Veerasammy, John M. Freund, Liara M. Gonzalez

**Affiliations:** Department of Clinical Sciences, College of Veterinary Medicine, North Carolina State University, 1060 William Moore Drive, Raleigh, NC 27607 USA

**Keywords:** Horse, Stem cell, 3D culture, Organoid, Enteroid, Intestine, Storage

## Abstract

**Background:**

Equine intestinal epithelial stem cells (ISCs) serve as potential targets to treat horses with severe intestinal injury. The ability to isolate and store ISCs from intestinal biopsies creates an opportunity for both in vitro experiments to study ISC dynamics in a variety of intestinal diseases, and, in the future, utilize these cells as a possible therapy. If biopsies could be successfully stored prior to processing for ISCs, this would increase the availability of sample repositories for future experimental and therapeutic use. However, delayed culture of equine ISCs following prolonged sample storage has not been described. The objective of this study was to describe the isolation and culture of equine ISCs following delayed tissue storage. Small intestinal full thickness biopsies were collected post euthanasia. Fresh tissue was immediately processed or stored at 4 °C for 24, 48 and 72 h (H) before processing. Intestinal stem cells (crypts) were dissociated and cultured. Size, growth efficiency and proliferation potential were compared between resultant enteroids (“mini-guts”) derived from each storage timepoint. In a separate study, growth efficiency of cryopreserved crypts was compared to cryopreserved enteroid fragments to investigate prolonged storage techniques.

**Results:**

Intestinal crypts were successfully isolated and cultured from all timepoints. At 72H post initial collection, the intestine was friable with epithelial sloughing; resultant dissociation yielded more partial crypts. Enteroids grown from crypts isolated at 72H were smaller with less proliferative potential (bud units, (median 6.5, 3.75–14.25)) than control (median 25, 15–28, *p* < 0.0001). No statistical differences were noted from tissues stored for 24H compared to control. Following cryopreservation, growth efficiency improved when cells were stored as enteroid fragments (median 81.6%, 66.2–109) compared to crypts (median 21.2%, 20–21.5, *p* = 0.01). The main limitations included a small sample size and lack of additional functional assays on enteroids.

**Conclusions:**

Equine ISCs can be isolated and cultured after prolonged tissue storage. Resultant enteroids had minimal differences even after 24-48H of whole tissue storage. This suggests that ISCs could be isolated for several days from samples properly stored after procedures, including surgery or necropsy, and used to create ISC repositories for study or therapy of equine intestinal diseases.

## Background

Colic remains a leading cause of morbidity and mortality in horses [1]. Mortality increases when horses are diagnosed with strangulating forms of colic where decreased intestinal blood supply and oxygen result in compromise and loss of the protective epithelial barrier [2]. The epithelial barrier is renewed and maintained by the crypt-based intestinal epithelial stem cells (ISCs) [3]. In murine models of intestinal damage, ISCs have been shown to directly improve epithelial repair after injury [4, 5]. The isolation and culture of equine ISCs (as crypt units) into three-dimensional (3D) “mini-guts” (enteroids) has been described [6, 7]. Additional studies are needed in this area to determine if and how equine ISCs could serve as potential therapeutic targets to treat horses with severe intestinal injury.

The ability to collect, expand, and store ISCs creates the opportunity to utilize their regenerative potential to both study intestinal disease or be used as a potential therapeutic including for transplantation [8, 9]. Researchers in 2013 demonstrated that murine ISCs could be stored at 4 °C for up to 30 h (H) with comparable proliferative potential [10]. More recently, processed samples from human surgical biopsies and cadavers were stored for up to 144H in organ preservation solution and yielded both successful crypt isolation and ISC culture [11]. However, the culture of equine ISCs following delayed tissue storage has not been investigated. Given the possibility of equine intestinal sample collection from many geographical locations or following procedures such as surgery or necropsy to store ISCs for future therapeutic use, the authors sought to investigate the effect of a longer whole tissue sample storage time (up to 72H) on the subsequent culture of equine ISCs. Additionally, the authors wished to investigate the effect of cryopreservation on the growth potential of freshly isolated equine ISCs and determine if cell storage conditions could be improved for future application. Therefore, the objectives of this study were two-fold; first, to describe the isolation and culture of ISCs into 3D enteroids following delayed tissue storage of up to 72H and second, to compare the growth efficiency of ISCs cryopreserved immediately following tissue dissociation to those cryopreserved as enteroid fragments after initial culture.

## Results

### Histologic assessment of stored samples

**Fig. 1:**
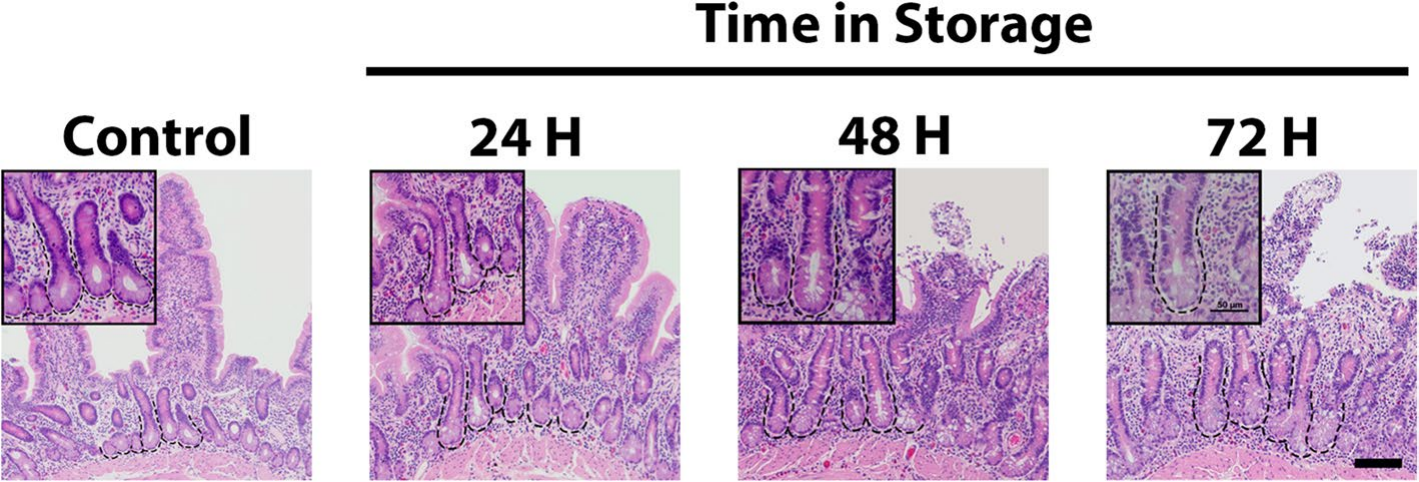
Histologic evaluation of representative equine small intestinal jejunum following delayed tissue storage. Tissue was processed immediately after collection or stored in phosphate buffered saline (PBS) and refrigerated at 4 °C for 24, 48 or 72 h (H). The epithelium remains largely intact following 24H of storage. Loss of the villus epithelium and villus sloughing is apparent at longer storage times. Scale bar *inset* 50 μm. *n* = 2–3. Scale bar panel 100 μm

Full thickness tissue samples were collected from all storage time points and processed for routine histomorphometric analysis. Samples from control tissue demonstrated intact epithelium along the intestinal villus and crypt (Fig. 1). At 24H, the villus epithelium remained largely intact with no obvious disruption of the ISC-rich crypts. As storage time increased, the epithelium continued to slough with significant loss seen by 72H.

### Growth efficiency and proliferative potential

**Fig. 2:**
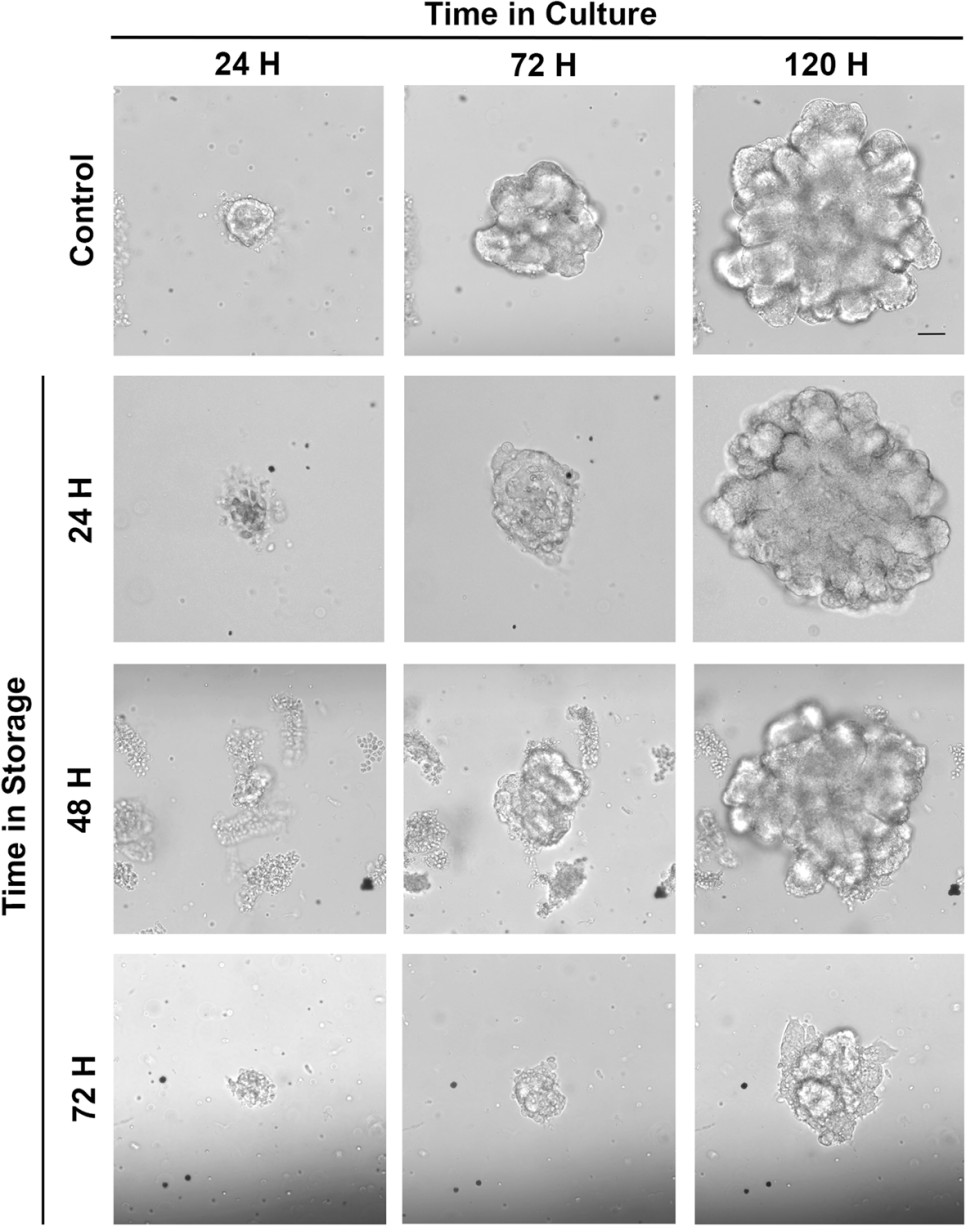
Growth of equine intestinal epithelial stem cells in culture following delayed sample storage. Intestinal stem cells (as crypt units) were successfully isolated and cultured from all storage time points. Proliferative, budding enteroids were seen in all groups by 120H. *n* = 3/group. Scale bar 50 μm

**Fig. 3:**
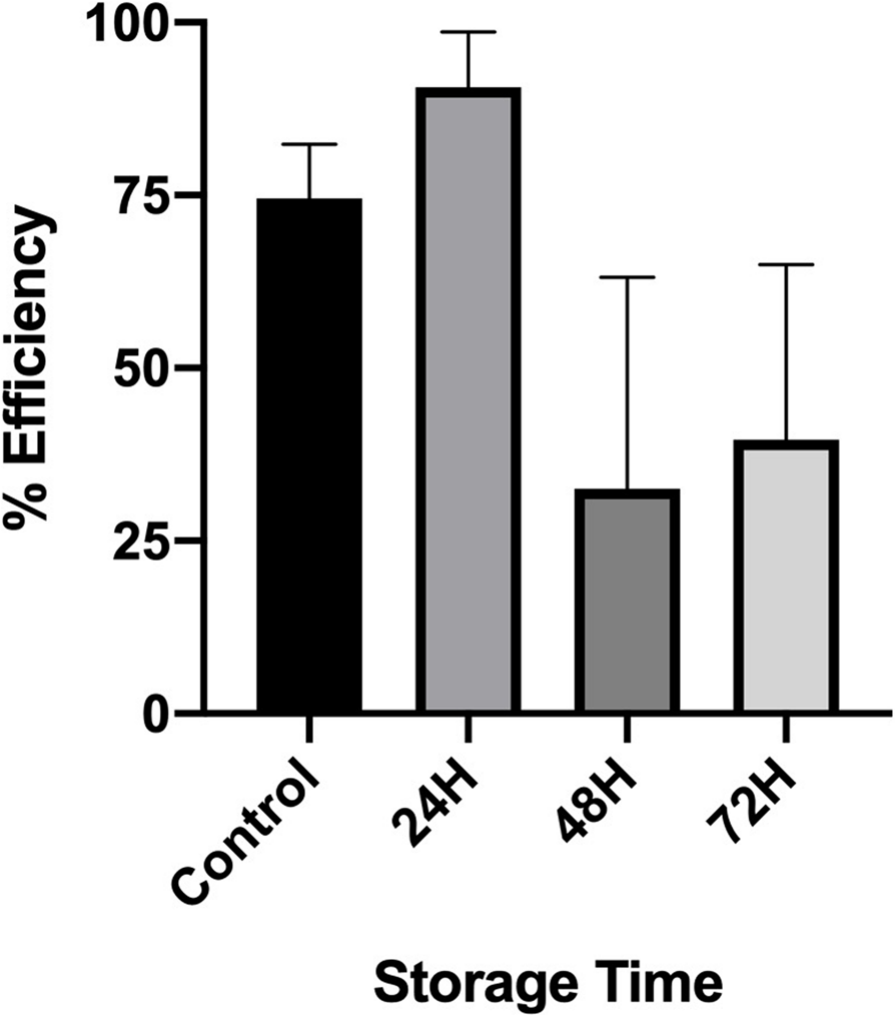
Median growth efficiency and range of crypt units following delayed sample storage. Following successful isolation and culture, no statistical difference was present in growth efficiency between storage groups (*p* = 0.08). *n* = 3–5/group

**Fig. 4:**
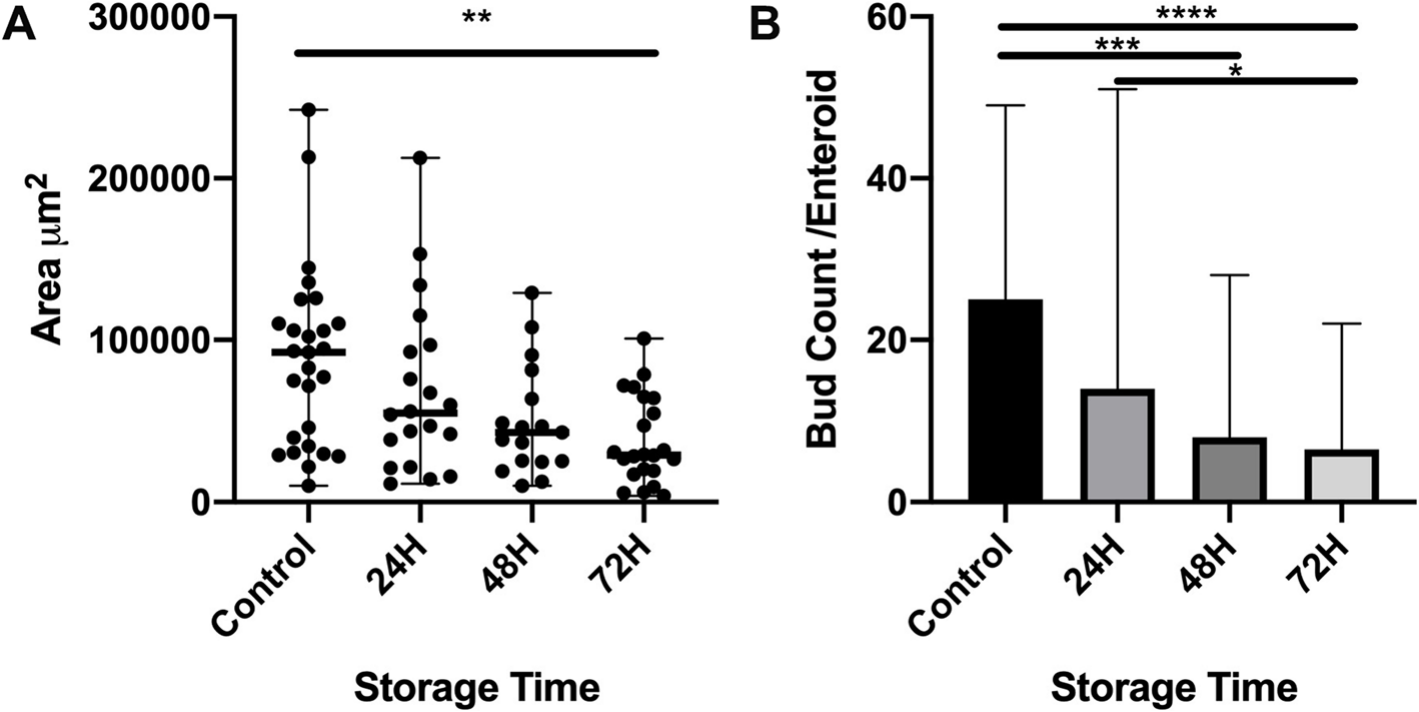
Median enteroid growth area and proliferation (bud count) after 5 days (120H) in culture. **A** Enteroids successfully cultured from tissue stored for 72H were significantly smaller than other groups (** *p* = 0.002). **B** Resultant enteroids from tissue stored for 48 and 72H were less proliferative than control and 24H (* *p* = 0.03, **p = 0.003, ****P < 0.0001). There was no difference in cultures resulting from tissue stored for 24H. 5–10 enteroids were measured per horse per timepoint, *n* = 3/group

Intestinal crypt units were successfully isolated from all storage timepoints, plated in culture and grown for 5 days (120H) as shown in Fig. 2. There was no statistical difference in the growth efficiency of plated crypts between control and storage timepoints, with median efficiencies and range of 74.5% (41.2–80.2), 90.6% (63–98.6), 32.5% (26.1–63.2) and 39.6% (33.8–64.9) from control, 24H, 48H and 72H of storage respectively (*p* = 0.08, Fig. 3). When comparing the overall size of mature enteroids, enteroids derived from crypts stored for 72H had significantly decreased area (median 29,132 µm; range 18,806–64,588 µm) when compared to control (median 92,372 µm; range 34,738–110,034 µm; *p* = 0.002, Fig. 4A). There was no statistical difference in overall area between enteroids grown from crypts stored for 24 or 48H and control. To determine the effect of storage time on proliferation and bud formation of resultant enteroids, the number of buds per enteroid was counted at 120H in culture (Fig. 4B). The use of budding and bud count as a measure of proliferation is well-documented [12–15]. As the duration of time in storage increased, the proliferation potential decreased with significantly less buds at 48H (median 8, range 4.5–19) and 72H (median 6.5, range 3.75–14.3) of storage when compared to control (median 25, range 15–28) (*p* = 0.003 and < 0.0001, respectively). Enteroids derived from tissue stored for 24H (median 14, range 9.5–28.5) were more proliferative than those stored for 72H (*p* = 0.03). There was no statistical difference between the proliferation potential of enteroids derived from either control or 24H stored tissue.

### Growth efficiency following cryopreservation

**Fig. 5:**
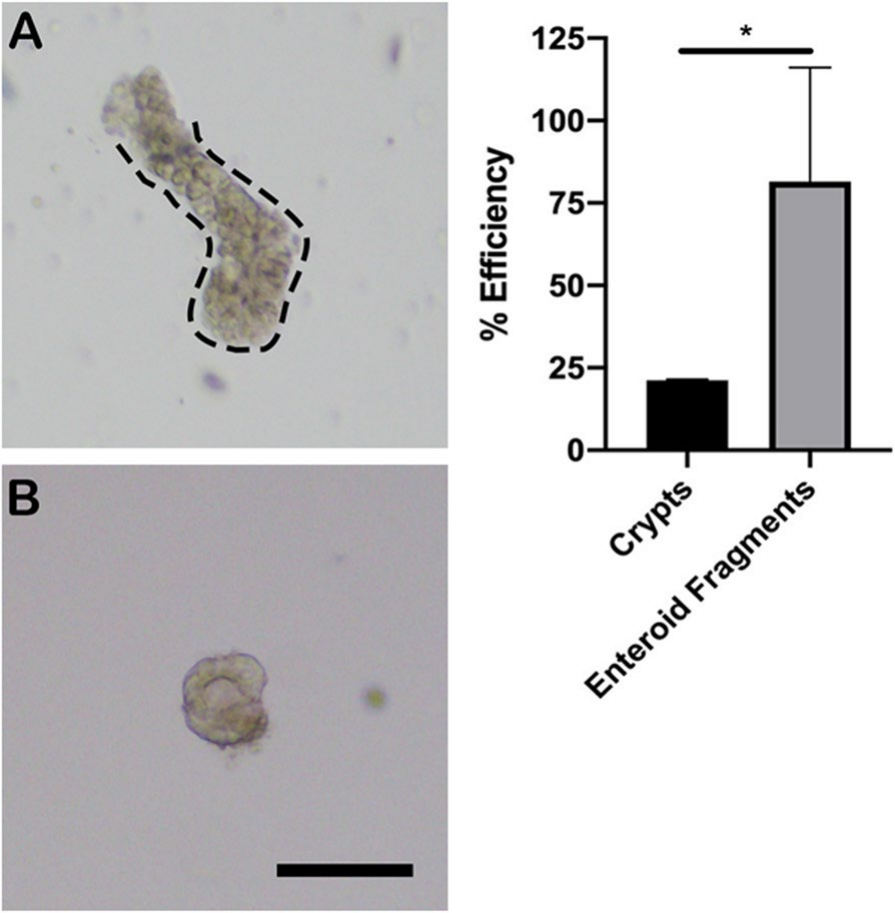
Growth efficiency of cryopreserved equine intestinal stem cells (as crypts) compared to frozen enteroid fragments. Cryopreserved crypts (**A**) and enteroid fragments (**B**) were thawed, plated and monitored in culture. Frozen fragments from previously grown enteroids had increased growth potential when plated in culture and compared to frozen crypts. * *p* = 0.01, *n* = 3–4. Scale bar 100 μm

Enteroids were successfully grown both from fresh crypt units cryopreserved immediately following tissue isolation and from enteroid fragments cryopreserved after growth in culture for 5–7 days. Cryopreservation of fresh, frozen crypts significantly decreased their resultant growth efficiency after 48H in culture (median 21.2%, range 20–21.5) when compared to cryopreserved enteroid fragments (median 81.6%, range 66.2–109) (*p* = 0.01; Fig. 5).

## Discussion

Equine crypt units containing ISCs were successfully isolated and grown in culture after samples underwent several days of whole tissue storage. This promotes the collection and storage of tissue following procedures including surgery or necropsy, and increases the likelihood that healthy, equine intestinal epithelial stem cells could be isolated and used to create ISC repositories. Based on the procedures performed in this experiment, ideal isolation of ISCs should occur within the first 24H of collection, however, samples could be stored up to 72H and successfully cultured. All intestinal samples should be stored in cold saline/phosphate buffered saline (PBS) at 4 °C until crypt isolation [10]. A recent study successfully cultured crypt units from human tissue stored up to 144 H and had improved yields and subsequent growth if tissue was stored in a specialized, commercially available preservation solution (University of Wisconsin/UW Solution) [11]. It is unlikely that the majority of equine practitioners would have quick access to these specific preservation solutions which is why the authors chose to store the tissue in more readily and widely available PBS. The delayed storage times selected in this paper were meant to model a variety of shipment times. Crypt units isolated from samples at all time points yielded enteroid growth. However, the overall proliferative potential, measured by the number of resultant bud units formed, decreased significantly as storage time increased. This likely occurs secondary to tissue disruption and cellular apoptosis prior to and during tissue dissociation, which results in the plating of smaller crypt units and single cells. Based on the second part of this study, the authors also recommend the cryopreservation of enteroid fragments over freshly isolated crypt units to increase future yield of enteroids, whether for future experiments, or to utilize as a possible therapeutic. To do this, it is recommended that enteroids be grown into complex, mature, multiple budding structures (at least 5–7 days to allow for differentiation; minimum 10–20 buds per enteroid) and then dissociated into smaller fragments prior to freezing [6]. Growth efficiency rates of 87–90% have been published following cryopreservation of murine enteroids for 7 days [12]. In the current study, cryopreserved crypt units and enteroids were stored for 7–24 months prior to assessing viability, further supporting the ability to bank these cells for future use.

Intestinal epithelial stem cells have now been isolated and cultured from a variety of veterinary species including the dog, cat, pig, cow and horse [6, 7, 16–22]. Researchers are utilizing enteroids as both a model for the study of zoonotic diseases such as Salmonella and Toxoplasma and to isolate stem cells from injured gastrointestinal tissues to better understand naturally occurring intestinal diseases [18, 21]. Future research is needed to determine the therapeutic potential of large animal ISCs, however murine studies are promising [4, 5].

One major limitation to this project is the lack of additional functional assessment of the enteroids following tissue storage. Immunohistochemistry staining for intestinal stem cells and post-mitotic cells could be performed and quantified between timepoints to determine if storage had an effect on stem cell number and differentiation. Assays to address epithelial cell ion transporters (including NHE3 or CFTR) have been documented and could be utilized to test additional cellular function [23]. Future work includes determining if there are functional differences between storage timepoints. Furthermore, tissues from each horse were not available for every experiment, limiting overall group size and subsequent analyses.

Overall, this project highlights the ability to collect and grow equine intestinal epithelial stem cells after several days in storage, a technique which can be applied broadly to improve cell culture in both human and veterinary species. Additionally, through the utilization of enteroid fragments, cryopreservation methods can be improved to yield significantly more cells for future experiments and, ideally, to be used as a future therapeutic for horses suffering from gastrointestinal disease.

## Conclusion

Equine ISCs can be isolated and cultured after prolonged tissue storage. Resultant enteroids had minimal differences even after 24-48H of whole tissue storage. This suggests that ISCs could be isolated for several days from samples properly stored after procedures, including surgery or necropsy, and used to create ISC repositories for study or therapy of equine intestinal diseases. Additionally, it was demonstrated that cryopreservation of equine ISCs as enteroid fragments yielded superior growth efficiency over cells stored as crypt units. Improved cellular efficiency is important to consider when maximizing the number of cells needed for future studies or as a possible therapeutic for transplantation.

## Methods

### Animals and sample collection

All animal use was approved by the Institutional Animal Care and Use Committee at North Carolina State University. Tissues were obtained from a total of 15 horses (12 geldings, 3 mares) that were not euthanized for this study but were free from overt signs of systemic disease, and euthanized for reasons unrelated to gastrointestinal disease such as orthopedic or behavioral conditions. Of note, at the time of euthanasia, several horses (*n* = 5) had been recently administered a non-steroidal anti-inflammatory (NSAID) medication. All jejunal tissue appeared grossly normal and was used in this study as reported adverse effects of NSAIDs are most often localized to the stomach and right dorsal colon [24]. Horses ranged from 5–15 years of age and included 4 Thoroughbreds, 2 Warmbloods, 2 Quarterhorses, 2 Arabians and 1 of each of the following: Saddlebred, Haflinger, Morgan, Draft and Paint. Our previous work demonstrated no correlation between age and resultant enteroid efficiency [6]. Immediately following euthanasia with pentobarbital sodium (Fatal-Plus, Vortech Pharmaceuticals, MI), several 20 cm long sections of mid-jejunum were removed through a celiotomy incision and placed into cold PBS. For storage studies, tissue sections were stored in PBS and placed into a 4 °C refrigerator for 24, 48 or 72 H prior to processing as described.

### Intestinal epithelial crypt isolation, enteroid culture, and analysis

Intestinal epithelial stem cells (ISCs, crypt units) were isolated as described previously [6]. Briefly, the excised jejunum was washed in PBS and opened longitudinally. The intestine was sectioned into smaller squares measuring 1–2 cm in diameter. Several small pieces of full thickness tissue were incubated for 30 min (min) in a 50 ml-conical tube with PBS containing 30 mM Ethylenediaminetetraacetic acid (EDTA), 10 mM Y-27632, 1 mM DTT, 1X antibiotic–antimycotic and Primocin (InvivoGen US, San Diego, CA). The conical tube was maintained on ice on an orbital shaking platform moving at 60 rpm in addition to vigorous shaking performed at 5 min intervals. Tissue pieces were transferred into a 37˚C pre-warmed PBS solution containing 30 mM EDTA, 10 mM Y-27632, 1X antibiotic–antimycotic and Primocin. The tissue was incubated in this solution at 37˚C for 10 min and shaken vigorously to help mobilize the crypt/villi units. Following this incubation, the tissues were placed in an ice-cold PBS wash containing 1X antibiotic–antimycotic for 5 min. Tissue was then transferred into additional washes and shaken until crypt/villi units were seen with minimal background debris as previously described [6]. Following the final wash, the remnant intestine was removed and the remaining solution was filtered using a 100-micron sterile cell strainer to remove the villi. A desired crypt yield of approximately 50 crypts/50 µL was determined by examining several 50 µL aliquots at 10X. The crypts were then pelleted in preparation to plate.

The pelleted crypts were re-suspended directly into a growth factor reduced extracellular matrix (Matrigel®, Corning®, Corning, NY) supplemented with 100 ng/mL recombinant human Noggin, 500 ng/mL recombinant human R-Spondin, 50 ng/mL recombinant human EGF, 100 ng/mL recombinant human Wnt3a, 10 mM Y-27632, 1 mM Nicotinamide, 10 nM Gastrin, 10 mM SB202190, 500 nM LY2157299 and 2.5 μM glycogen synthase kinase 3 inhibitor (GSK3i, CHIR99021). Between 25–50 crypt units were plated in 50 µL of Matrigel on a 24 well plate. After allowing the matrix to polymerize for 30 min at 37˚C, each well was overlaid with 500 mL of Advanced DMEM/F12 containing the supplements 1X N-2 supplement, 1X B-27 supplement minus vitamin A, 1X Glutamax, 100 mg/mL penicillin/streptomycin and 1 mM Hepes buffer. Growth factors were added to the media 48H after plating and subsequent 48H intervals. The entire volume of media was changed 96H following plating and every subsequent 96H interval.

Plating efficiency was determined by first counting the number of enterospheres or enteroids formed every 24H for 5 days (120H) and then dividing the total count by the starting number of crypt units initially plated within each Matrigel matrix.

Intestinal stem cell rich bud units were counted per enteroid after 120H in culture to determine proliferative potential as each bud is capable of independently forming a new enteroid [12]. For each timepoint, 5–10 enteroids were counted per horse per storage group.

### Cryo-preservation of equine crypts and enteroid fragments

To assess the effect of freezing on growth efficiency of crypts, freshly isolated equine jejunal crypts were suspended in 1 ml freeze-preservation medium consisting of 10% dimethylsulfoxide (DMSO) in 1X DMEM/F12 culture medium with no growth factors and were frozen slowly over 12-18H using a cryo freezing container (Mr. Frosty ™, Thermo Fisher Scientific, Waltham, MA), containing isopropanol placed in a -80 ˚C freezer. This method is designed to achieve an optimum cooling rate for cell preservation of close to -1 °C/minute. Following removal from the -80 ˚C freezer, tubes were stored in liquid nitrogen. As previously described, cryopreservation tubes were obtained from liquid nitrogen storage and warmed in a 37˚C water bath to thaw [6]. The tubes were centrifuged to pellet the crypts at 200G for 5 min. The freezing media was removed and the crypt pellet was resuspended in cooled Matrigel and plated as above on a 24 well plate [6]. Efficiency was counted after 48 H in culture as described above.

To assess the effect of freezing on growth efficiency of enteroid fragments, mature, complex enteroids grown in culture for 120-168H were split using aggressive pipetting techniques and suspended in freeze preservation medium as described above. Fragments were then thawed and plated. Efficiency of resultant enteroids was calculated at 48H as described above.

### Measurements and statistics

One author (ASS) was responsible for all enteroid measurements and cell counting. Enteroid area was measured using the “Measurement and ROI” feature from the Olympus micro imaging software cellSens (Olympus Corporation, Tokyo, Japan). Enteroids were identified at 24H post plating, tagged, and followed over a period of 120H. Five to ten enteroids were measured per horse at each storage time point. All measurements were performed in triplicate and averaged for each experiment. Statistical analysis was performed using GraphPad Prism 8 (GraphPad Software, San Diego, CA). Outliers were identified using ROUT analysis. Samples were assessed for normality using the Shapiro–Wilk test and found to not be normally distributed. Subsequent nonparametric analysis was performed using the Kruskal–Wallis test with *p* < 0.05 significant and subsequent post-hoc analysis performed using Dunns multiple comparisons. Growth efficiency of cryopreserved crypt units compared to enteroid fragments was analysed using a Welch’s t-test. Based on our previous growth data, to achieve a power of 0.8, a sample size of 4 horses was required to detect a significant difference between sample means.

## Data Availability

All data generated and analysed during this study are included in this published article. Data are available from the first (Amy Stieler Stewart, alstiele@ncsu.edu) and final author (Liara M. Gonzalez, lmgonza4@ncsu.edu) upon reasonable request.

## Abbreviations

ISCs: Intestinal epithelial stem cells
PBS: Phosphate buffered saline
EDTA: Ethylenediaminetetraacetic acid
